# Anti-cancer and potential chemopreventive actions of ginseng by activating Nrf2 (NFE2L2) anti-oxidative stress/anti-inflammatory pathways

**DOI:** 10.1186/1749-8546-5-37

**Published:** 2010-10-27

**Authors:** Constance Lay-Lay Saw, Qing Wu, Ah-Ng Tony Kong

**Affiliations:** 1 Center for Cancer Prevention Research, Ernest Mario School of Pharmacy, Rutgers, the State University of New Jersey, USA; 2 Department of Pharmaceutics, Ernest Mario School of Pharmacy, Rutgers, the State University of New Jersey, USA; 3 Department of Pharmaceutics, School of Chinese Materia Medica, Beijing University of Chinese Medicine, Beijing, China

## Abstract

This article reviews recent basic and clinical studies of ginseng, particularly the anti-cancer effects and the potential chemopreventive actions by activating the transcriptional factor, nuclear factor (erythroid-derived 2)-like 2 (Nrf2 or NFE2L2)-mediated anti-oxidative stress or anti-inflammatory pathways. Nrf2 is a novel target for cancer prevention as it regulates the antioxidant responsive element (ARE), a critical regulatory element in the promoter region of genes encoding cellular phase II detoxifying and anti-oxidative stress enzymes. The studies on the chemopreventive effects of ginseng or its components/products showed that Nrf2 could also be a target for ginseng's actions. A number of papers also demonstrated the anti-inflammatory effects of ginseng. Targeting Nrf2 pathway is a novel approach to the investigation of ginseng's cancer chemopreventive actions,  including some oxidative stress and inflammatory conditions responsible for the initiation, promotion and progression of carcinogenesis.

## Background

Ginseng protects the cardiovascular system, stimulates the central nervous system [1] and possesses anti-cancer activities [2,3] inhibiting human gastric adenocarcinoma [4] and human breast carcinoma [5]. Therefore, ginseng is a potential cancer preventive agent [6].

Nuclear factor (erythroid-derived 2)-like 2 (Nrf2 or NFE2L2) is a key regulator of the antioxidant responsive element (ARE)-mediated gene expression and therefore a potential anti-cancer target for chemopreventive compounds [7], including ginseng [8-10]. However, concerns have been raised for possible inappropriate claims of ginseng products [11,12]. This article reviews the potential chemopreventive actions of ginseng *via *the Nrf2 signalling pathway and the potential molecular mechanism of ginseng's anti-cancer effects.

### Literature search

A full literature search (up to 2010) was conducted during November 2009 till April 2010 with 'ginseng' as the search keyword was performed in PubMed and the Chinese National Knowledge Infrastructure (CNKI). Other keywords used in the search included 'ginseng', 'Nrf2', 'chemoprevention', 'cancer prevention', 'clinical studies' and 'anti-cancer'. A total of 3917 and 147 papers from PubMed and CNKI respectively were retrieved and screened for anti-cancer clinical studies with ginseng. Seven published articles were found in PubMed with the keywords 'ginseng' and 'Nrf2' including a paper on *Angelica sinensis *(*Danggui*) [13].

### Ginseng in Chinese medicine

In Chinese medicine, a disorder is often a manifestation of an imbalance between yin and yang and/or changes in the pathogenic and antipathogenic *qi*. Ginseng is the drug of choice for replenishing *qi*, especially in the case of *qi *collapse. Major Chinese medicinal uses of ginseng and its commercial products and their indications are provided in Table 1.

**Table 1:** Use of ginseng in Chinese medicine

Actions	Indications	Chinese medicine products
To replenish the primordial *qi*	Collapse due to prostration of primordial *qi*, exhibiting extremely cold limbs, sweating, vertigo and shortness of breath.	*Shenfu tang *- decoction of ginseng and *Aconiti Lateralis*; *Sinijia renshen tang *- decoction of ginseng for treating yang exhaustion.
To nourish the spleen and stomach	Spleen and stomach *qi *deficiency complicated by dampness, exhibiting weakness of limbs, emaciation, indigestion, vomiting or diarrhoea, fullness in the epigastrium and chest.	*Shenlingbaizhu san *- powder of ginseng, *Poria *and *Atractylodis Macrocephalae*; *Jianpi wan *- pills for strengthening the spleen.
To promote the production of body fluid	Impairment of both *qi *and yin, exhibiting lassitude, shortness of breath, excessive perspiration, dry throat, thirst; also for long-standing cough due to lung deficiency.	*Shengmai yin *- liquid of ginseng, *Radix Ophiopogonis *and *Fructus Schisandrae *for restoring the pulse.
To invigorate the spleen and nourish the heart	Disorders involving heart and spleen deficiency, exhibiting palpitation, amnesia, insomnia, poor appetite, fatigue; also for cases of hemafecia, metrorrhagia and metrostaxis.	*Guipi wan *- ginseng pills for invigorating spleen and nourishing the heart; *Renshen yangrong wan *- pills of ginseng for nourishing *qi *and yin.

### Clinical studies on ginseng as adjuvant therapy for cancer

Ginseng possesses preventive and therapeutics effects on cancer [14,15]. Ginseng is used to treat cancer or to reinforce the effects and/or reduce the side effects of chemotherapy [16,17]. Ginseng polysaccharides and ginsenosides are the main ingredients contributing to anti-cancer action of ginseng [18-21]. Ginseng boosts the patient's immunity, suppresses the proliferation of tumour cells, inhibits the formation of new blood vessels in tumours, induces apoptosis of tumour cells, anti-metastasis of tumour and immunomodulation [3,6]. Additional file 1 lists recent clinical studies of ginseng products as adjuvant therapy to chemotherapy and radiotherapy in China [22-27].

### Significance of the Nrf2-ARE signalling pathway in cancer chemoprevention

**Figure 1:**
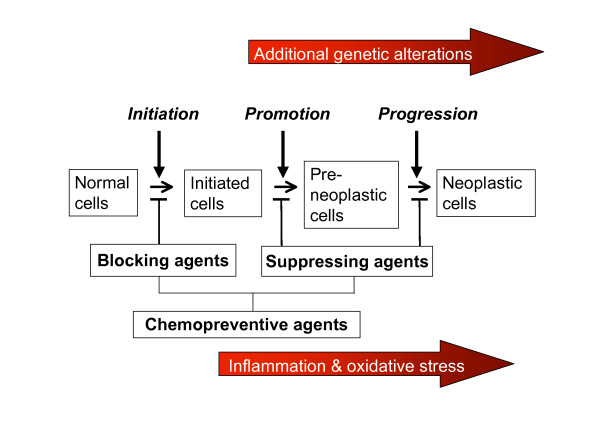
**Carcinogenesis is a multiple steps process**. The initiation step is started by the transformation of the normal cell into a cancer cell (initiated cell). These cells undergo tumour promotion into preneoplastic cells, which progress to neoplastic cells. Inflammation and oxidative stress, together with the accumulation of genetic alterations over a lifetime of patients, will result in the formation of cancer. It is important to take note that in reality, cancer may arise without proceeding through each of these steps. Chemopreventive agents can interfere with different steps of this process. Some agents inhibit metabolic activation of the procarcinogens to their ultimate electrophilic species, or their subsequent interaction with DNA. These agents therefore block tumour initiation (blocking agents). Alternatively, blocking agents can stimulate the detoxification of carcinogens, leading to their excretion from the body. Other agents suppress (suppressing agents) the later steps (promotion and progression). Some agents can act as both blocking and suppressing agents.

Carcinogenesis involves multiple steps including transition of normal cells to pre-initiated cells and ultimately invasive carcinoma, providing ample opportunities for chemoprevention. In general, tumour development follows three distinct yet closely interrelated phases (I-III), namely initiation, promotion and progression [28,29]. When cells are exposed to oxidative stress, DNA may go through oxidative damage [30] coupled with persisting inflammation [31] as well as formation of DNA adducts, leading to increased genomic instability, enhanced neoplastic transformation and ultimately cancer (Figure 1). Various cancer chemopreventive compounds, including natural dietary and synthetic compounds, are found to be effective in preventing cancer formation at all of these three developmental stages [32-34] (Figure 1). Curcumin is one of such natural dietary chemopreventive compounds with promising findings from clinical trials [35,36]. When the cell is exposed to oxidative stress such as reactive oxygen species (ROS), reactive nitrogen species (RNS) or carcinogenic species, induction of phase I, phase II and phase III enzymes/transporters occur [37,38]. Carcinogens are typically metabolized *via *oxidation and reduction by phase I enzymes [39]. The resulting products are conjugated with endogenous cofactors such as glutathione (GSH) by glutathione *S*-transferase (GST), a phase II enzyme forming water soluble products that can be easily excreted [39,40]. Induction of other phase II enzymes such as uridine-diphospho-glucuronosyltransferases (UGT) may also enhance the excretion of carcinogens such as heterocyclic aromatic amines, the well-known genotoxic chemicals formed during preparation of foods [41,42].

**Figure 2:**
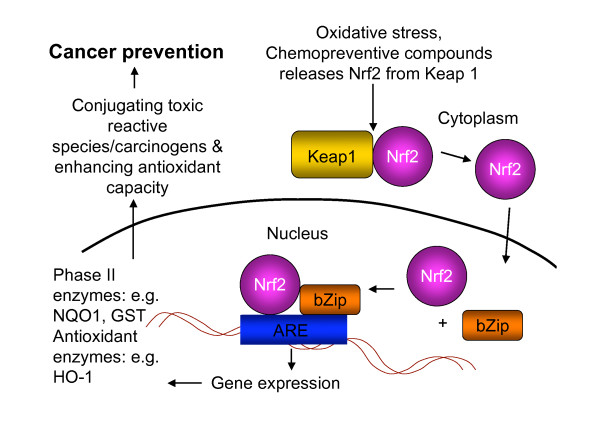
**Schematic presentation of Nrf2-ARE pathway**. In the cytoplasm, under basal level, newly synthesized Nrf2 is constitutively bound to Keap1 forming a dimer, Nrf2-Keap1. Keap1 is a cytosolic protein that inhibits Nrf2 signalling by promoting Nrf2 degradation through proteasomal pathway. When oxidants such as ROS, RNS and dietary chemopreventive compounds react with redox reactive cysteines in Keap1, Nrf2 will be released from Keap1, hence allowing the transcriptional factor Nrf2 to translocate to the nucleus. In the nucleus, Nrf2 dimerizes with basic leucine zipper partners (bZip) such as small MAF-family proteins and bind to ARE, which is located in the promoter of the phase II and antioxidative genes, triggering the transcription of ARE-regulated genes. The critical role of Nrf2 in protecting cells/subjects from neoplastic transformation when subject to oxidative stress and carcinogens is performed by enhancing the expression of detoxifying metabolizing enzymes and maintaining oxidative stress homeostasis by producing antioxidant enzymes. Application of chemopreventive compounds can further enhance the expression of phase II detoxifying and antioxidant enzymes by up-regulating the Nrf2-ARE expression.

The induction of phase II enzymes can be attributed to the transcriptional control of the ARE by Nrf2 [7]. Nrf2 is a key regulator of ARE-mediated gene expression and a potential target for chemopreventive compounds [43-45]. Nrf2 is inhibited in the cytoplasm by the anchor protein Kelch-like ECH-associated protein-1 (Keap1), a cytosolic protein that inhibits Nrf2 signalling by promoting Nrf2 degradation through proteasomal pathway [46]. In the presence of oxidative stress or chemical inducers, Nrf2 is released from Keap1 inhibition, translocates to the nucleus and binds to ARE consensus sequences [47]. Activation of Nrf2 by chemopreventive agents influences the expression of phase II and anti-oxidative stress enzymes such as heme oxygenase 1 (HO-1) [48]. HO-1 catalyzes the degradation of heme to carbon monoxide, iron and biliverdin and is thought to be essential in cellular defensive mechanisms and is implicated in various pathophysiological conditions such as inflammation, atherosclerosis, neurodegenerative diseases and cancers [49]. Since the first isolation of Nrf2 in 1995, the function of Nrf2 has been studied widely [50]. It appears that the most important role of Nrf2 is activating the ARE-mediated anti-oxidative responses [47]. The current understanding of the molecular Nrf2-ARE pathway is illustrated in Figure 2 as a schematic presentation of the proposed mechanism by which ARE and its downstream target enzymes are induced upon transcriptional activation [7,47,51,52]. Under normal physiological conditions, ROS and other endogenous reactive molecules are also constantly being produced during normal aerobic metabolism, based on numerous experimental evidence, such constitutive gene expression is also thought to be under the regulation of ARE by Nrf2 [47]. Interestingly, many chemopreventive compounds, including ginseng, are inducers of ARE. Additional file 2 lists the studies of ginseng and its extract [53-56] in activating the Nrf2-ARE pathway.

### Anti-oxidative and anti-inflammatory effects of ginseng

Kim *et al. *reported that ginseng extract induced the elevation of catalase and superoxide dismutase activities in sedentary male patients [57]. Another study reported significant reduction of oxidative stress biomarkers such as F2-isoprostane and 8-hydroxy-deoxyguanosine in healthy patients after oral administration of ginsenoside-enriched *Panax quinquefolius *(American ginseng) extract [58]. As the study did not measure specific enzymes, it is not clear whether the reduction of these markers was due to the induction of antioxidant enzymes. As an *in vivo *study found that ginsenosides induced cytochrome (CYP) P450 1A1 which plays an important role in xenobiotic metabolism as well as in carcinogenesis [59], the drug interactions between ginseng and conventional drugs including chemotherapeutic agents should be recognized. It was postulated that ginsenoside competed with aryl hydrocarbons for both the aryl hydrocarbon receptor and CYP1A1, which may explain ginseng's chemopreventive properties [59]. Another study reported that a water extract of ginseng inhibited benzo[a]pyrene (BaP)-induced hepatotoxicity and CYP1A1 expression and reversed the reduction of GSH content and GST activities induced by BaP in rats [8]. Moreover, various isoforms of phase II gene GSTs were significantly induced by the ginseng extract *via *activating the Nrf2-ARE pathway. Therefore, the latter *in vivo *study [8] showed great promise for future studies of ginseng and chemoprevention in chemical-induced animal carcinogenesis models.

**Figure 3:**
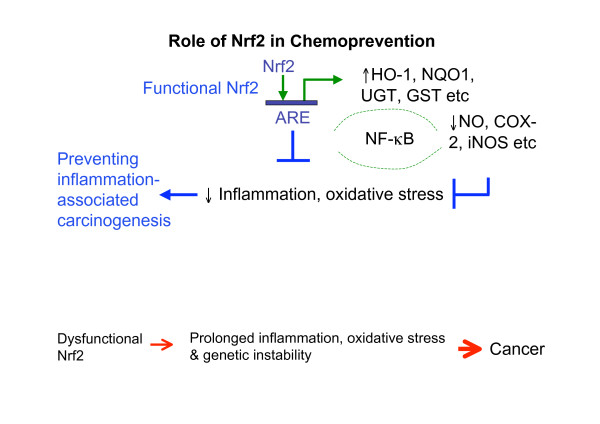
**A simplified illustration showing the role of Nrf2 in anti-oxidative and anti-inflammatory pathways preventing carcinogenesis**. Upon stimulation by ROS, RNS (having negative effects in subjects) and chemopreventive compounds (having positive effects in subjects), Nrf2 is activated and NF-kB pathways can also be mediated concurrently, such multiple interactions allow chemopreventive compounds, including ginseng, to exert their beneficial cancer preventive and therapeutic effects.

The role of Nrf2 is not only implicated in the induction of the antioxidant and phase II genes, but is also involved in anti-inflammation. One of the key transcriptional factors in inflammatory response is the nuclear factor-kappa-B (NF-kB) and many chemopreventive compounds have been reported that those compounds work through activating the Nrf2 pathway also suppressing inflammatory activities [44,60-64]. Glutathione peroxidase 2 (Gpx2) prevented the exacerbation of inflammation induced by cyclooxygenase-2 (COX-2) expression and inflammation driven initiation of carcinogenesis [65]. Various ginsenosides inhibited inducible nitric oxide synthase (iNOS)-induced NO production [66] and down-regulated COX-2 expression [67]. Interestingly, ginseng induced the expression of γ-glutamylcystein ligase (γ-GCL) and enhanced production of GSH in ginsenoside Rd treated hepatocytes [68]. One would expect that Nrf2 would be induced by ginsenoside Rd as well, however, it was reported that ginsenoside Rd increased the nuclear level of p65, which is the subunit of NF-kB complex, but not the level of Nrf2 [68]. Such observation is rather uncommon, as other reports have shown that ginsenosides are suppressing NF-kB which will be presented below. Therefore, effects of ginsenoside Rd on NF-kB pathway warrants additional detailed experiment for verification. One of the metabolite of ginsenoside, 20(S)-protopanaxatriol inhibited iNOS and COX-2 expressions through inactivation of NF-kB [69]. Evidence supports the notion that blocking NF-kB is an important target for the control of inflammation and cancer [70,71]. The interplays between Nrf2 and NF-kB signalling pathways were studied in our laboratory with a bioinformatics approach [72] and with an Nrf2-/- mouse model [61,73]. We found potential common members involved in the crosstalk between Nrf2 and NF-kB signalling pathways, such as some of the upstream mitogen-activated protein kinases (MAPKs). Increasing evidence supports the existing crosstalk between Nrf2 pathway and anti-inflammation [61,73-77]. It is likely that some of the components in ginseng targeting the Nrf2 pathway and enhancing the expression of ARE-mediated antioxidant and phase II genes would suppress the aberrant inflammatory responses regulated by the NF-kB pathway concomitantly (Figure 3).

### Future studies

In future studies, it would be important to correlate the oxidative stress markers and the development of oxidative stress induced-diseases such as cancer in chemopreventive studies using ginseng/ginseng products. Properly designed long-term clinical studies should be performed to investigate the chemopreventive activities of ginseng, particularly the Nrf2-related antioxidant and phase II detoxifying enzymes as many cancer patients worldwide, have been using ginseng for boosting the immunity or general well-being during chemotherapy, radiotherapy or post-surgery.

## Conclusion

The anti-cancer and chemopreventive actions of ginseng could be exerted through activating the Nrf2 anti-oxidative and anti-inflammatory pathways. Further studies on the effects of ginseng in Nrf2-mediated induction of phase II/antioxidant enzymes would elucidate the action mechanism of ginseng in cancer chemoprevention.

## Abbreviations

Nrf2 (NFE2L2): Nuclear factor (erythroid-derived 2)-like 2; ARE: Antioxidant responsive element; CNKI: Chinese National Knowledge Infrastructure; ROS: Reactive oxygen species; RNS: Reactive nitrogen species; GSH: Glutathione; GST: Glutathione *S*-transferase; UGT: Uridine-diphospho-glucuronosyltransferases; Keap1: Kelch-like ECH-associated protein-1; HO-1: Heme oxygenase 1; CYP: Cytochrome; BaP: Benzo[a]pyrene; NF-kB: Nuclear factor-kappa-B; Gpx2: Glutathione peroxidase 2; COX-2: Cyclooxygenase-2; iNOS: Inducible nitric oxide synthase; γ-GCL: γ-glutamylcystein ligase; MAPKs: Mitogen-activated protein kinases; bZip: Basic leucine zipper partners; GSP: Ginseng polysaccharides; KPS: Karnofsky Performance Status Scale; NPC: Nasopharyngeal carcinoma; RT: Radiotherapy; NK: Natural killer; LAK: Lymphocyte activated killer; NQO1: NADPH: quinone oxidoreductase 1; AKR: Aldo-keto reductases.

## Competing interests

The authors declare that they have no competing interests.

## Authors' contributions

CLLS planned this review. CLLS and QW performed the literature searches and drafted the manuscript. ANTK supervised the review process and revised the manuscript. All authors read and approved the final version of the manuscript.

## Supplementary Material

Additional file 1 **Clinical studies of ginseng Chinese medicine products as adjuvant therapy to cancer treatments**. Click here for file

Additional file 2 **Preclinical studies on ginseng and its extracts showing molecular activities on Nrf2 activation for potential chemopreventive use**. Click here for file
