# Supplementary material for: Anti-cancer and potential chemopreventive actions of ginseng by activating Nrf2 (NFE2L2) anti-oxidative stress/anti-inflammatory pathways
Source: Chin Med. 2010 Oct 27;5:37. doi: 10.1186/1749-8546-5-37 (PMC2990743; doi:10.1186/1749-8546-5-37)
Supplement: Additional file 1 — Clinical studies of ginseng Chinese medicine products as adjuvant therapy to cancer treatments. [file 1749-8546-5-37-S1.DOC]

Additional file 1: Clinical studies of ginseng Chinese medicine products as adjuvant therapy to cancer treatments

| **Ginseng Chinese medicine product** | **Type of cancer** | **Clinical investigation design** | **Results** | **References** |
| --- | --- | --- | --- | --- |
| Ginseng polysaccharides (GSP) injection | Non-small cell lung carcinoma | Combined with routine chemotherapy (COM or MOF) | Karnofsky Performance Status Scale (KPS) was improved significantly in patients received GSP (*P*<0.05). The side effects of chemotherapy in patients was reduced with GSP as compared to those who did not, neutropenic incidence was also reduced significantly (*P*<0.05). | [22] |
|  | Ovarian cancer | Combined with routine chemotherapy | Compared with the control group, the physical conditions of patients in the treatment group were significantly improved (*P*<0.05), by KPS assessment, and the side effects of chemotherapy, such as leukopenia, were reduced (*P*<0.05). GSP was found to be non-toxic and did not increase liver and renal toxicities. | [23] |
|  | Nasopharyngeal carcinoma (NPC) | Combined with Radiotherapy (RT) | Clinical examination conducted 3 months after the treatment showed that the complete remission rate in the RT-GSP group was 96.6%, and 93.3% in the control group, the complete remission rate of cervical node metastatic tumor in the two groups was 85.7% and 78.0% respectively. The activity of natural killer (NK) cell and lymphocyte activated killer (LAK) cell as well as T3, T4 value in peripheral blood increased significantly in the RT-GSP group (all *P*<0.05) after treatment, while in the control group, activity of NK cell and LAK cell changed insignificantly after treatment, and T3, T4 value lowered significantly (*P*<0.05). No toxic-adverse reaction of GSP was found and GSP has shown to improve immune function in NPC patients during RT. | [24] |
| *Shenyi* capsules (ginsenoside Rg3) | Breast cancer | Combined with routine chemotherapy (CAF or CMF) | The symptoms of *qi*-deficiency in patients of the test group improved significantly as compared to the control group (*P*<0.05 or *P*<0.01). The level of T cell subtype function (CD4/CD8) increased significantly in the test group compared with the control group (*P*<0.001). | [25] |
|  | Advanced non-small cell lung cancer | Combined NP regime | Ginsenoside Rg3 capsules combined NP regimen is effective in the treatment of advanced non-small cell lung cancer, and ginsenoside Rg3 capsules decreased the occurrence of leucopenia (*P*<0.05), improved KPS scale (*P*<0.05) and the cytoimmunity function (*P*<0.05). | [26] |
| *Shengmai* injection (ginseng, *Radix Ophiopogonis*, *Fructus Schisandrae*) | 223 cases of different kinds of cancer | Combined with routine chemotherapy | KPS scale of test group patients increased significantly more than that of the control group (*P*<0.01). | [27] |
