# Supplementary material for: Anti-cancer and potential chemopreventive actions of ginseng by activating Nrf2 (NFE2L2) anti-oxidative stress/anti-inflammatory pathways
Source: Chin Med. 2010 Oct 27;5:37. doi: 10.1186/1749-8546-5-37 (PMC2990743; doi:10.1186/1749-8546-5-37)
Supplement: Additional file 2 — Preclinical studies on ginseng and its extracts showing molecular activities on Nrf2 activation for potential chemopreventive use. [file 1749-8546-5-37-S2.DOC]

Additional file 2: Preclinical studies on ginseng and its extracts showing molecular activities on Nrf2 activation for potential chemopreventive use

| **Testing model/method** | **Study design/testing compounds** | **Outcome effects** | **References** |
| --- | --- | --- | --- |
| A coupled tetrazolium dye reduction assay | Korean red ginseng extract, protopanaxatriol, panaxytriol | Induction of NADPH: quinone oxidoreductase 1 (NQO1). | [9],[53] |
| Optical method for monitoring the activities of ARE dependent genes | Panaxytriol | Induction of aldo-keto reductases (AKR) in human neuroblastoma IMR-32 cells (AKR1C3) and human liver HepG2 cells (AKR1C2). | [54] |
| Protection on 6-hydroxydopamine induced oxidative stress | Ginsenoside Rb1 | Induction of Nrf2-ARE-HO-1 pathway in human neuroblastoma SH-SY5Y cells | [10] |
| Protection on benzo[a]pyrene induced hepatotoxicity in rats | Water extract from wild ginseng (*Panax ginseng* C.A. Meyer) | Reductions in GSH content and GST activity were reversed by the ginseng extract. Expression of GSTA2, GSTA3 and GSTM2 were significantly increased by the extract via the Nrf2-ARE pathway. | [8] |
| Protection on polychlorinated biphenyls induced oxidative stress | Korean red ginseng extract | Induction of Nrf2-ARE-HO-1/GCL in rat pheochromocytoma cells (PC12). | [55] |
| *In vitro* and *in vivo* studies: Protection on angiogensin II/tumor necrosis factor alpha/hydrogen peroxide induced oxidative stress/cell death in rat cardiomyocytes H9C2 cells; oral gavage of American ginseng extract in mice | Standardized American ginseng extract | Induction of Nrf2 protein expression, nuclear translocation, transcriptional activity, direct binding to its target gene promoters and expression of Nrf2-mediated anti-oxidative genes in H9C2 cells; protection on the oxidative stress-induced cell death in H9C2 cells.  Induction of Nrf2, NQO1, HO-1 and thioredoxin reductase-1 in murine heart. | [56] |
